# The Neural Correlates of Mindful Awareness: A Possible Buffering Effect on Anxiety-Related Reduction in Subgenual Anterior Cingulate Cortex Activity

**DOI:** 10.1371/journal.pone.0075526

**Published:** 2013-10-09

**Authors:** Yuko Hakamata, Mikio Iwase, Takashi Kato, Kohei Senda, Toshiya Inada

**Affiliations:** 1 Department of Clinical Psychology, Graduate School of Medical Sciences, Kitasato University, Sagamihara, Kanagawa, Japan; 2 Koseikai Hospital Diagnostic Imaging Center, Toyohashi, Aichi, Japan; 3 Department of Clinical and Experimental Neuroimaging, Center for Development of Advanced Medicine for Dementia, National Center for Geriatrics and Gerontology, Obu, Aichi, Japan; 4 Department of Psychiatry, Seiwa Hospital, Institute of Neuropsychiatry, Shinjuku, Tokyo, Japan; Banner Alzheimer’s Institute, United States of America

## Abstract

**Background:**

Human personality consists of two fundamental elements character and temperament. Character allays automatic and preconceptual emotional responses determined by temperament. However, the neurobiological basis of character and its interplay with temperament remain elusive. Here, we examined character-temperament interplay and explored the neural basis of character, with a particular focus on the subgenual anterior cingulate cortex extending to a ventromedial portion of the prefrontal cortex (sgACC/vmPFC).

**Methods:**

Resting brain glucose metabolism (GM) was measured using [^18^F] fluorodeoxyglucose positron emission tomography in 140 healthy adults. Personality traits were assessed using the Temperament and Character Inventory. Regions of interest (ROI) analysis and whole-brain analysis were performed to examine a combination effect of temperament and character on the sgACC/vmPFC and to explore the neural correlates of character, respectively.

**Results:**

Harm avoidance (HA), a temperament trait (i.e., depressive, anxious, vulnerable), showed a significant negative impact on the sgACC/vmPFC GM, whereas self-transcendence (ST), a character trait (i.e., intuitive, judicious, spiritual), exhibited a significant positive effect on GM in the same region (HA *β* = −0.248, *p* = 0.003; ST: *β* = 0.250, *p* = 0.003). In addition, when coupled with strong ST, individuals with strong HA maintained the sgACC/vmPFC GM level comparable to the level of those with low scores on both HA and ST. Furthermore, exploratory whole-brain analysis revealed a significant positive relationship between ST and sgACC/vmPFC GM (peak voxel at x = −8, y = 32, z = −8, *k* = 423, *Z* = 4.41, corrected *p* ^FDR^ = 0.030).

**Conclusion:**

The current findings indicate that the sgACC/vmPFC might play a critical role in mindful awareness to something beyond as well as in emotional regulation. Developing a sense of mindfulness may temper exaggerated emotional responses in individuals with a risk for or having anxiety and depressive disorders.

## Introduction

Substantial individual differences exist in emotional, cognitive, and behavioral responses to the environment, i.e., personality. When the responses become exaggerated and cause significant distress, psychopathological changes occur. Personality consists of two key elements: temperament and character. Temperament evokes automatic and preconceptual responses of emotion particularly, whereas character curbs the responses initiated by temperament through conceptual organization [1], [2]. Although both elements are influenced by biological factors, such as brain function, temperament is more directly susceptible due to its instinctive nature and closely associated with emotional dysregulation [2]. For example, harm avoidance (HA), one of the temperament dimensions measured with the Temperament and Character Inventory (TCI), determines emotional reactivity to potential danger or punishment [1]. Several longitudinal studies suggest that individuals who possess strong HA are significantly predisposed to chronic anxiety disorders and major depression in comparison with those who do not [3]–[6]. Thus, exploring the neurobiological interplay between HA and character may provide new insights for the prevention or treatment of anxiety and depressive disorders.

Previous neuroimaging studies on personality exclusively focused on the neural basis underlying temperament to understand the pathophysiology of emotional dysregulation. As the critical site that molds HA, a subgenual portion of the anterior cingulate cortex, which extends to the ventromedial prefrontal cortex (sgACC/vmPFC), has attracted much attention. It has been shown, in magnetic resonance imaging (MRI) studies, that individuals with strong emotional reactivity to possible punishment have smaller gray matter volume in the sgACC/vmPFC [7]–[9], although the opposite has also been reported [10]–[13]. Similarly, functional imaging studies using positron emission tomography (PET) revealed an association between HA and activity in the sgACC/vmPFC during resting state, which reflects mental activity patterns under the primary influence of an individual’s personality. In previous studies of the TCI temperament dimensions, we demonstrated that greater HA is a reliable predictor of lower glucose metabolism (GM) in the sgACC/vmPFC [14], a finding which supports previous reports of a negative relationship between HA and sgACC activity [15], [16], [17]. In line with these findings, sgACC/vmPFC activity has been shown to be significantly decreased in patients with anxiety and major depressive disorders [18]–[21]. Altogether, these findings suggest that attenuated activity in the sgACC/vmPFC might be a risk factor for emotional dysregulation.

On the contrary, evidence on the correlation between neural activities and character is limited. Character consists of cooperativeness (C): identification with and acceptance of others manifested as altruism, self-directedness (SD): self-determination and willpower to accomplish a person’s own goal, and self-transcendence (ST): identification with everything conceived as essential and consequential parts of a unified whole. A few PET studies employing preset regions of interest (ROI) have reported a significant positive association between SD and dopamine synthesis capacity in the bilateral caudate nucleus [22] and a significant negative correlation between ST and serotonin 1A receptor (5HTR1A) binding potentials (BP) in the raphe nuclei, hippocampus, and neocortex [23]. In addition, a significant correlation between C and extensive areas in the fronto-temporo-parietal cortices was found in some MRI studies [24], [25]. Given that character diminishes negative emotion caused by HA [1], [2], character might alleviate the negative impact of HA on the sgACC/vmPFC by enhancing its decreased activity. To our best knowledge, no neuroimaging study has considered the interplay between these different personality elements.

In this study, we used PET with 2-fluoro-2-deoxy-d-glucose (FDG) to investigate the relationships between personality traits (temperament and character) and resting GM in a large sample that was completely independent from those used in our previous studies [14], [15]. We especially focused on the sgACC/vmPFC and on a combination effect of HA and a character dimension on this region. First, we confirmed a significant negative correlation between HA and the sgACC/vmPFC GM using an ROI-based analysis. Next, we examined whether character, in contrast to HA, could be a significant positive predictor of the sgACC/vmPFC GM. When a character dimension was found to parallel the sgACC/vmPFC GM, the coupling effect of HA and this character dimension on this regional GM was investigated. Furthermore, we explored the neural basis of character on cerebral brain GM using an exploratory whole-brain analysis, considering an interaction between character and HA.

## Methods

### Ethical Considerations

This study was approved by the Ethics Committee of Seiwa Hospital, Institute of Neuropsychiatry, and the Koseikai Hospital Advanced Imaging Center. After receiving a full description of the study, each participant provided written informed consent. All the research procedures were conducted in accordance with the Helsinki Declaration.

### Participants

Participants were recruited from a population of healthy individuals who had undergone medical examination at the Koseikai Hospital Advanced Imaging Center (Toyohashi, Japan). Individuals with Axis I psychiatric disorders or a history of substance abuse, defined according to the Structured Clinical Interview of the *Diagnostic and Statistical Manual of Mental Disorders*, Fourth Edition (DSM-IV) [26], or neurological or significant medical illness, such as brain tumor, atrophy, or lacunar infarct detected during the preceding MRI, were excluded from the study.

### Personality Trait Assessment

Personality traits were assessed using the 125-item version of the TCI [1], which includes temperament and character dimensions. Temperament consists of novelty seeking (NS): frequent exploratory activity in response to novelty, reward dependence (RD): sensitivity to feelings and approval of others, persistence (P): continuing and preserving of behavior despite fatigue and lack of reward, and HA. Character consists of SD, C, and ST as mentioned earlier. Each item was evaluated on a four-point Likert scale (from completely disagree: 1 to strongly agree: 4).

### Positron Emission Tomography Procedures and Analysis

PET images were acquired using the Discovery ST Elite Performance system (in-plane resolution: 4 mm full width at half maximum; 47 coronal slices of 3.27 mm; GE Healthcare, Wisconsin, USA). Participants fasted for 6 h before receiving intravenous injection of 185 MBq ^18^F-FDG. The PET scan was initiated 40 min after injection, and continued for 10 min in a dimly lit room with no photic or auditory stimulation. During both injection and scanning, subjects were asked to lie supine with their eyes closed and rest their minds.

For whole-brain analysis, spatial preprocessing was performed with the Statistical Parametric Mapping (SPM) 8 software (Institute of Neurology, University College London, London, UK) [27]–[29]. All reconstructed images were spatially normalized to the Montreal Neurological Institute standard template in order to remove intersubject anatomical variation [30]. Affine transformation was performed in order to determine 12 optimal parameters to register the brain on our original FDG template. Subtle differences between the transformed image and the template were removed by a nonlinear registration method, using the weighted sum of the predefined smooth basis functions used in discrete cosine transformation. Spatially normalized images were smoothed by convolution with an isotropic Gaussian kernel, with 8-mm full width at half maximum.

For the ROI analysis, the relative (proportional scaling) sgACC/vmPFC GM values were extracted for each participant using MarsBar version 0.43 (http://marsbar.sourceforge.net/) [31], following the definitions of previous meta-analytic studies on the sgACC and vmPFC [32], [33] (sgACC: box center at x = 7/−8, y = 26, z = −6, volume = 10×10×10 mm; vmPFC: box center at x = 7/−8, y = 40, z = −18, volume = 14×14×14 mm) (**Figure S1**).

### Statistical Analyses

To confirm a negative correlation between HA and sgACC/vmPFC GM, partial correlation coefficients were calculated between HA and GM in the above-mentioned ROIs, controlling for age and sex. To examine whether the character dimensions (i.e., SD, C, and ST) have significantly impact on the sgACC/vmPFC GM, a step-wise multiple regression analysis was performed on average GM level of the sgACC/vmPFC regions that showed significant correlation with HA. The 3 character dimensions as well as HA were incorporated in the model as independent variables, controlling for age, sex and the other temperament dimensions (NS, RD and P). Once a significant predictor was found in the analysis, a combination effect of HA and this trait on the sgACC/vmPFC GM was investigated. To control for age, sex, and the remaining 5 traits, an analysis of covariance (ANCOVA) was performed on the average sgACC/vmPFC GM among 4 different groups: individuals with >50% scores on HA but <50% scores on the character trait; those with >50% scores for both traits; those with <50% scores for both traits; and those with <50% scores on HA but >50% scores on the character trait. These statistical analyses were conducted using the SPSS version 19.0, and the two-tailed probability level was set at *p*<0.05. Age and sex were controlled in all analyses, given that cerebral GM, especially in the prefrontal area, is decreased in females and older population [34], [35].

In addition, we examined effects of character and an interaction between character and HA on cerebral brain GM by the exploratory whole-brain analysis. A multiple regression analysis was performed with age, sex, and all personality traits as covariates using the SPM 8. In creating each SPM contrast to show the results of significant relationship between cerebral GM and character dimensions, age, sex, and the temperament dimensions were set as nuisance effects (i.e., 0). For a character dimension that yielded a significant impact on a specific regional GM, an interaction effect with HA was further investigated on this region, controlling for age, sex, and the other dimensions. The resulting SPM (*t*) value was converted to an SPM (*Z*) value. The height threshold (*u*) was set at *p*<0.05, with cluster level correction for multiple comparisons (false discovery rate [FDR]). The extent threshold (*k*) was set at 100 voxels.

## Results

This study included 140 healthy participants (79 men and 61 women). Their mean age was 49.4 (±8.9 SD) years with a range from 26 to 67. Partial correlation coefficients between personality traits, controlled for age and sex, are shown in **Table S1**.

### Relationship between Harm Avoidance and Glucose Metabolism in the Subgenual Anterior Cingulate Cortex and Ventromedial Prefrontal Cortex

Partial correlation analysis confirmed a significant negative correlation between HA and GM in the right sgACC and bilateral vmPFC. The correlation for the sgACC and vmPFC on the right side remained significant after Bonferroni correction (*r* = −0.244, *p* = 0.004; *r* = −0.241, *p* = 0.004, respectively) (**Table 1**). We, therefore, used the average GM in these 2 regions as a dependent variable in the subsequent analyses.

**Table 1:** Partial correlations between harm avoidance and glucose metabolism in the subgenual anterior cingulate cortex and ventromedial prefrontal cortex (*N* = 140).

	HA	R sgACC	L sgACC	R vmPFC	L vmPFC
HA	–				
R sgACC	−***0.244*** **	–			
L sgACC	−0.086	***0.724*** ***	–		
R vmPFC	−***0.241*** **	***0.303*** ***	0.191*	–	
L vmPFC	−0.169*	***0.288*** ***	***0.283*** ***	***0.752*** ***	–

Controlled for age and sex. Degrees of freedom = 136.

* *p*<0.05,

** *p*<0.01,

*** *p*<0.001.

***Bold italic values***: Coefficients survived Bonferroni correction (*p*<0.013).

HA, harm avoidance; sgACC, subgenual anterior cingulate cortex; vmPFC, ventromedial prefrontal cortex; R, right; L, left.

ROI definitions: sgACC: box center at x = 7/−8, y = 26, z = −6, volume = 10×10×10 mm; vmPFC: box center at x = 7/−8, y = 40, z = −18, volume = 14×14×14 mm.

The definitions were based on the previous studies (32, 33).

### Relationship between Character Dimensions and Glucose Metabolism in the Subgenual Anterior Cingulate Cortex and Ventromedial Prefrontal Cortex

The step-wise multiple regression analysis on the GM in the right sgACC/vmPFC, with the 3 character dimensions and HA as independent variables, revealed that HA and ST were optimal for the prediction of average GM level in the right sgACC and vmPFC [*F*(7,132) = 7.935, *R^2^* = 0.544, *adjusted R^2^* = 0.259, *p*<0.001). The model explained 26% of the variance observed, and each predictor significantly affected the right sgACC/vmPFC GM (HA: *β* = −0.248, *p* = 0.003; ST: *β* = 0.250, *p* = 0.003) (**Table 2**).

**Table 2:** Stepwise multiple regression analysis for glucose metabolism in the right anterior cingulate cortex and ventromedial prefrontal cortex (*N* = 140).

Optimal model	*R^2^*	*adjusted R^2^*	*F*	*p*
	0.544	0.259	7.935	p<0.001
Selected independent variable		*standardized β*	*t*	*p*
Harm avoidance		−0.248	−3.003	0.003
Self-transcendence		0.250	2.982	0.003

Controlled for age, sex, and the temperament dimensions. Degrees of freedom (7,132).

Dependent variable was the average glucose metabolism level of the right subgenual anterior cingulate cortex and ventromedial prefrontal cortex.

ROI definitions: the right anterior cingulate cortex: box center at x = 7, y = 26, z = −6, volume = 10×10×10 mm; right vmPFC: box center at x = 7, y = 40, z = −18, volume = 14×14×14 mm.

These definitions were based on the previous studies (32, 33).

### A Combination Effect of Harm Avoidance and Self-transcendence on Glucose Metabolism in the Subgenual Anterior Cingulate Cortex and Ventromedial Prefrontal Cortex

**Figure 1:**
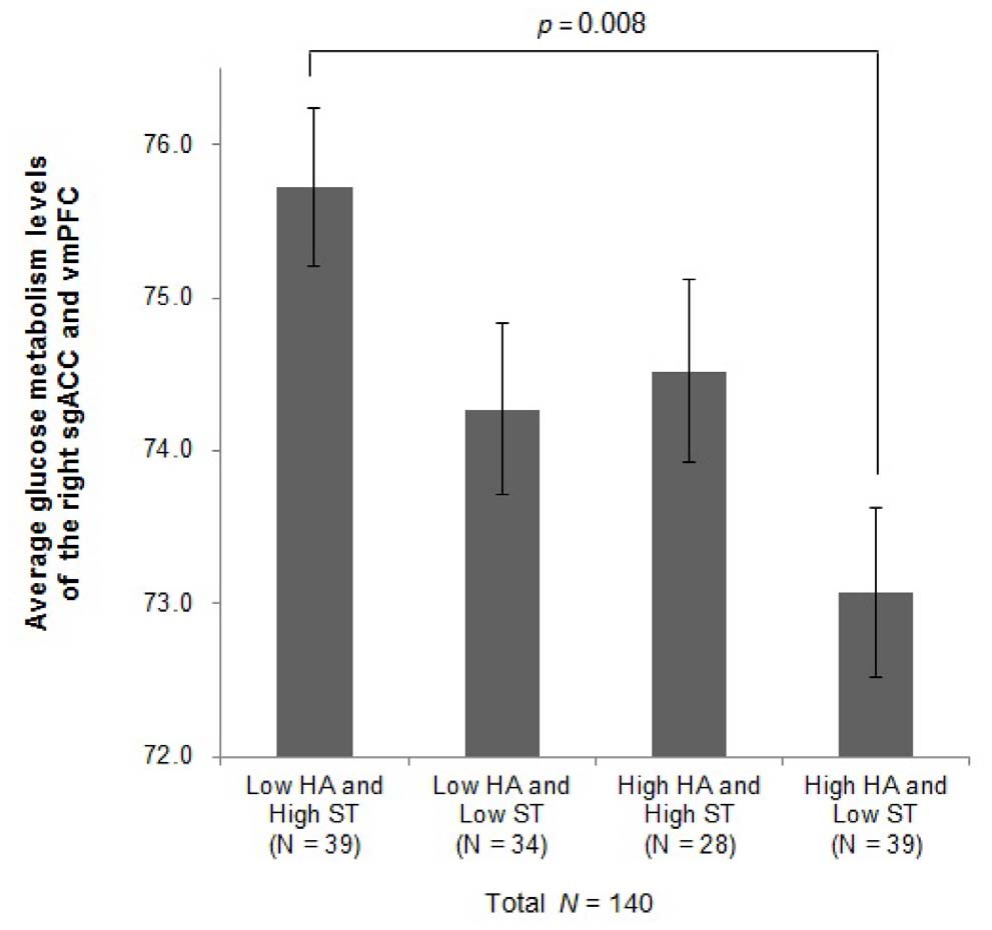
Plot of the average levels of glucose metabolism in the sgACC/vmPFC in 4 groups of participants with specific combinations of HA and ST. sgACC, subgenual anterior cingulate cortex; vmPFC, ventromedial prefrontal cortex; HA, harm avoidance; ST, self-transcendence. Error bars indicate standard errors of mean.

Since ST was a significant positive predictor of the right sgACC/vmPFC GM as well as HA, we further performed ANCOVA to investigate the combination effect of HA and ST on the average GM level in the right sgACC and vmPFC. We first created the following 4 groups: (i) individuals with <50% scores on HA but >50% scores on ST (*N* = 39); (ii) those with <50% scores on both HA and ST (*N = *34); (iii) those with >50% scores on both HA and ST (*N* = 28); and (iv) those with >50% scores on HA but <50% scores on ST (*N* = 39). ANCOVA demonstrated that the main effect of group was indeed significant, *F* (3, 129) = 3.800, partial *η* ^2^ = 0.081, *p* = 0.012. Post hoc analysis using Bonferroni’s criterion for significance indicated that Group i (*M* = 75.96, *SD* = 3.14) had significantly higher GM in the right sgACC/vmPFC in comparison to Group iv (*M* = 73.35, *SD = *3.35, *p* = 0.008). We did not find any significant difference between the other groups. The average GM level of each group was shown in **Figure 1**.

### Effects of Character and an Interaction between Character and Harm Avoidance on Cerebral Glucose Metabolism: Whole-brain Analysis

**Figure 2:**
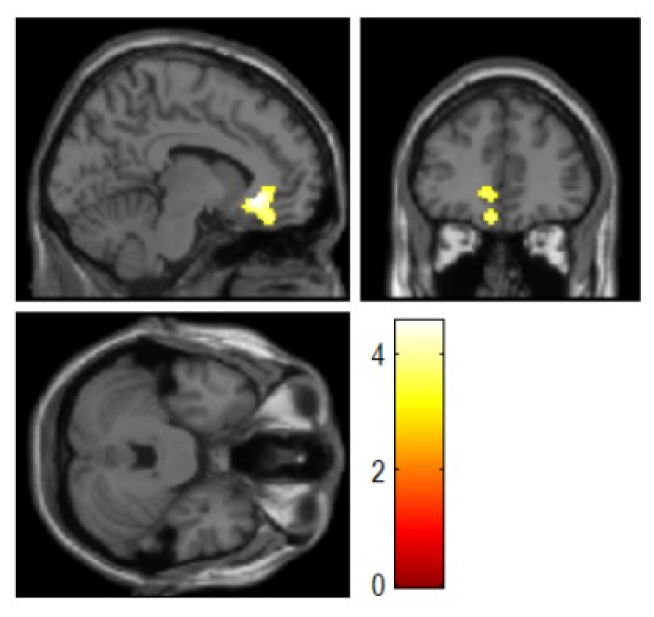
The subgenual anterior cingulate cortex encompassing a ventromedial portion of the prefrontal cortex that showed a significant positive correlation with self-transcendence (*N* = 140). Peak voxel at x = −8, y = 32, z = −8, *k* = 423, *Z* = 4.41, corrected *p* ^FDR^ = 0.030.

**Figure 3:**
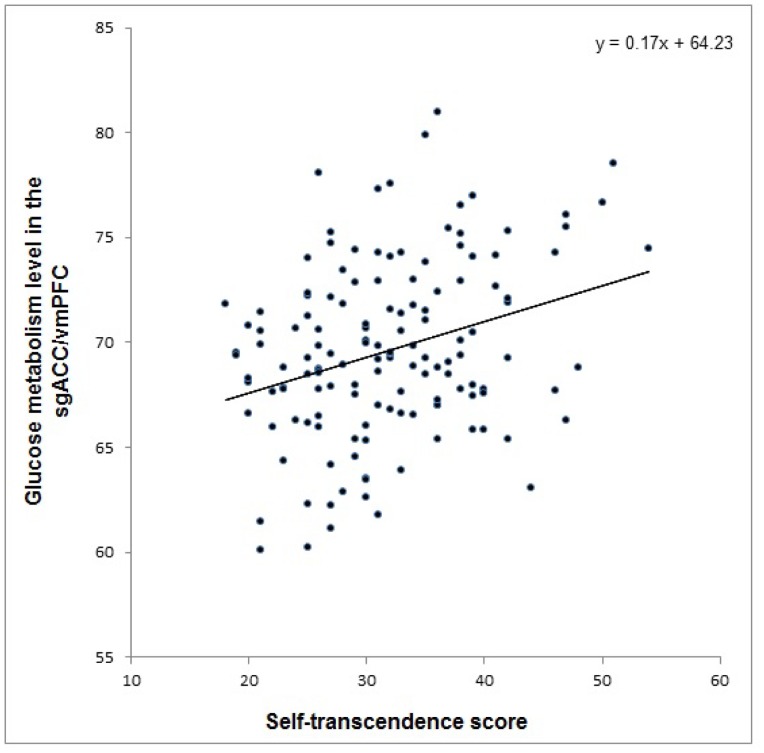
Scatter plot of the positive correlation between self-transcendence and glucose metabolism in the sgACC/vmPFC (*N* = 140). sgACC, subgenual anterior cingulate cortex; vmPFC, ventromedial prefrontal cortex. Peak voxel at x = −8, y = 32, z = −8, *k* = 423, *Z* = 4.41, corrected *p* ^FDR^ = 0.030.

The whole-brain analysis revealed a significant positive correlation between ST and the left sgACC/vmPFC GM (peak voxel at x = −8, y = 32, z = −8, *k* = 423, *Z* = 4.41, corrected *p* ^FDR^ = 0.030) (**Figure 2**). Scatter plot of the relationship between GM in this cluster and ST was shown in **Figure 3**. No significant correlation with other character dimensions was found. Further, as shown in the ROI analysis, the interaction effect of ST and HA demonstrated a significant impact on the sgACC/vmPFC GM at a more conservative significance level, indicating that individuals with a combination of higher ST and lower HA scores have greater GM levels in this region (peak voxel at x = 0, y = 22, z = −6, *k* = 651, *Z* = 4.16, corrected *p* ^FDR^ = 0.003).

## Discussion

Our main hypothesis was that character dimensions, which are theoretically presumed to harness emotional responses evoked by HA, would have a modulatory effect on HA-related GM reduction in the sgACC/vmPFC. Indeed, ROI analysis revealed that ST exerted a significant positive influence on the right sgACC/vmPFC GM that was negatively correlated with HA, indicating that ST might enhance the decreased sgACC/vmPFC activity in individuals who possess strong emotional reactivity to threats. Furthermore, in line with the results of ROI analysis, the exploratory whole-brain analysis revealed a significant positive association between ST and sgACC/vmPFC GM, whereas the other character dimensions failed to show any significantly associated regions. This is, to the best of our knowledge, the first neuroimaging study to show that the sgACC/vmPFC is involved in mindful awareness to something beyond (i.e., ST) as well as in emotional regulation.

With regard to HA, our data are consistent with previous studies showing a significant negative correlation between HA and the right sgACC/vmPFC, although there are some controversial reports on its laterality. It has been demonstrated that the right [10]–[12], [14], [16], [17], [36] and left [8], [9], [13], [15] sgACC/vmPFC are significantly associated with emotional reactivity to potential danger. The right side of the sgACC/vmPFC might have a relatively strong impact on HA, however, both sides might contribute to the formation of HA, given the strong correlation observed between each hemisphere (**Table 1**: *r* = 0.724, *p*<0.001 for the right and left sgACC; *r* = 0.752, *p*<0.001, for the right and left vmPFC) [19], [37].

In contrast, the neurobiological basis for ST is poorly understood at present. To our knowledge, so far, 2 PET studies have been conducted to investigate the relationship between ST and resting brain activity. In a study on 15 healthy subjects, a significant negative correlation between ST and 5HTR1A BP was found in the hippocampus, raphe nuclei, and neocortex [23]. It should be noted that the neocortex was defined as a large ROI in this study. The other study, on the contrary, failed to show a correlation between ST and 5HTR1A BP in 23 patients with major depression and in 20 healthy volunteers [38]. Additionally, patients with brain damage in the inferior posterior parietal regions showed a significant increase in ST scores after the ablation surgery [39], although patients with lesions in the sgACC/vmPFC were not included in this study.

For lesions in the sgACC/vmPFC, other line of studies have reported that patients with damage in this region exhibit a loss of self-insight and a marked reduction in specific types of negative affect, such as shame, guilt, embarrassment, and regret, which are more complicated than basic emotions, like anger or fear [40]–[43]. Moreover, the sgACC/vmPFC has been implicated in the integration of sensory information into self-relevant representations that result in self-insight [44]–[46]. Given that the sgACC/vmPFC constitutes extensive neural networks among the fronto-temporo-parietal cortices, including the hippocampus and the inferior parietal regions [39], [47], the sgACC/vmPFC might play a crucial role in sensory integration or fusion of self and transcendent entities as well. Yet, further research is clearly needed to elucidate the neural basis of ST.

Most importantly, a significant combination effect of HA and ST on the sgACC/vmPFC GM was observed in this study. The finding that no significant group difference was found between individuals with high scores on both HA and ST and those with low scores on both HA and ST, suggests that ST might offset the negative impact of HA on sgACC/vmPFC activity. In fact, ST can powerfully explain psychological well-being, such as a sense of happiness, in comparison to the other character dimensions (C and SD) [48], [49]. Thus, enhancing a sense of mindfulness and connection with something beyond can improve psychological well-being, even in people with a tendency to experience exaggerated emotional responses.

Several limitations should be considered when interpreting the current results. Firstly, we investigated cerebral GM during the resting state, which represents complicated mental processes [50]. To reveal the exact function of the sgACC/vmPFC, brain activity during a task designed to specifically target the region should be measured. Secondly, we did not examine network connectivity. In future studies, the neural basis of character dimensions should be further explored. Despite these limitations, the sample size in our study was larger than that in any previous PET study on personality traits.

In summary, the present results suggest that the sgACC/vmPFC might play a critical role in generating feelings and states of humanness and benevolence, in addition to emotional reactivity to potential punishment. Developing a sense of mindfulness and connection with something beyond may temper strong emotional reactivity to potential threats and protect against the development and persistence of anxiety and depressive disorders.

## Supporting Information

Figure S1 **Regions of interest: the subgenual anterior cingulate cortex and ventromedial prefrontal cortex.** Red color: subgenual anterior cingulate cortex; Blue color: ventromedial prefrontal cortex. The definition of regions of interest was based on previous studies (32, 33). Sagittal plane image presented at x = 7.
(TIF)

Table S1 **Partial correlations between personality traits (** ***N*** **= 140).**
(DOCX)
