# Supplementary figures and images for: The Neural Correlates of Mindful Awareness: A Possible Buffering Effect on Anxiety-Related Reduction in Subgenual Anterior Cingulate Cortex Activity
Source: PLoS One. 2013 Oct 9;8(10):e75526. doi: 10.1371/journal.pone.0075526 (PMC3794017; doi:10.1371/journal.pone.0075526)

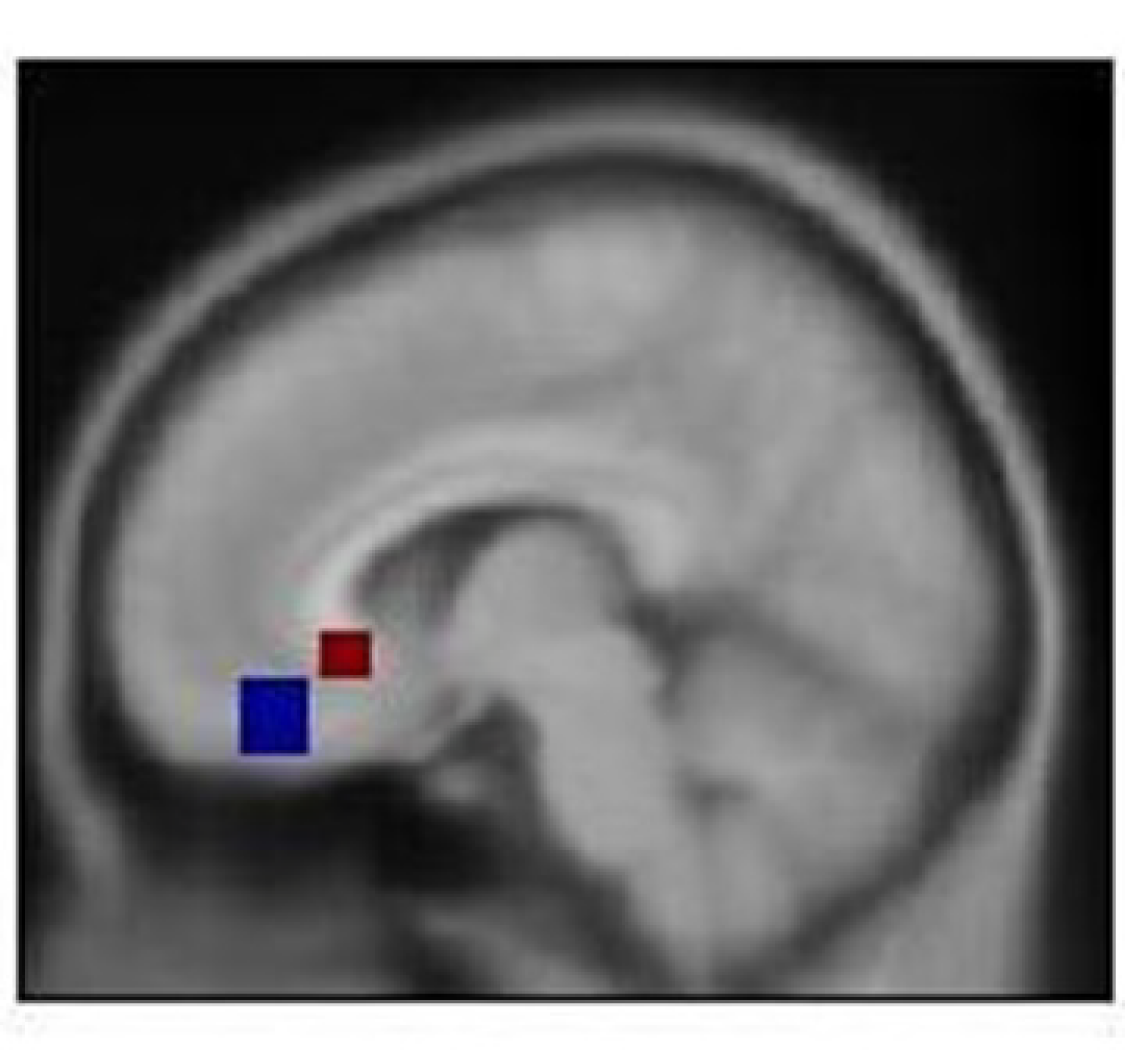

Supplement: Figure S1 — Regions of interest: the subgenual anterior cingulate cortex and ventromedial prefrontal cortex. Red color: subgenual anterior cingulate cortex; Blue color: ventromedial prefrontal cortex. The definition of regions of interest was based on previous studies (32, 33). Sagittal plane image presented at x = 7. (TIF) [file pone.0075526.s001.tif]
