# Supplementary material for: The Neural Correlates of Mindful Awareness: A Possible Buffering Effect on Anxiety-Related Reduction in Subgenual Anterior Cingulate Cortex Activity
Source: PLoS One. 2013 Oct 9;8(10):e75526. doi: 10.1371/journal.pone.0075526 (PMC3794017; doi:10.1371/journal.pone.0075526)
Supplement: Table S1 — Partial correlations between personality traits ( N = 140). (DOCX) [file pone.0075526.s002.docx]

| **Supplementary table S1. Partial correlations between personality traits (*N* = 140)** | | | | | | | | | | | | | | |
| --- | --- | --- | --- | --- | --- | --- | --- | --- | --- | --- | --- | --- | --- | --- |
|  | NS | | HA | | RD | | P | | SD | | C | | ST | |
| Novelty seeking (NS) | - |  |  |  |  |  |  |  |  |  |  |  |  |  |
| Harm avoidance (HA) | ***-0.238*** | * | - |  |  |  |  |  |  |  |  |  |  |  |
| Reward dependence (RD) | 0.122 |  | 0.098 |  | - |  |  |  |  |  |  |  |  |  |
| Persistence (P) | -0.094 |  | ***-0.323*** | ** | 0.040 |  | - |  |  |  |  |  |  |  |
| Self-directedness (SD) | -0.011 |  | ***-0.632*** | ** | 0.078 |  | ***0.267*** | * | - |  |  |  |  |  |
| Cooperativeness (C) | -0.101 |  | -0.159 |  | ***0.368*** | ** | ***0.260*** | * | 0.125 |  | - |  |  |  |
| Self-transcendence (ST) | 0.221 | * | ***-0.237*** | * | ***0.259*** | * | ***0.338*** | ** | 0.070 |  | ***0.320*** | * | - |  |
| Controlled for age and sex. Degrees of freedom = 136. **p* < 0.01, ***p* < 0.001. | | | | | | | | | | |  |  |  |  |
| ***Bold italic values***: Coefficients survived Bonferroni correction (*p* < 0.007) | | | | | | | |  |  |  |  |  |  |  |
